# Specular Reflections Detection and Removal for Endoscopic Images Based on Brightness Classification

**DOI:** 10.3390/s23020974

**Published:** 2023-01-14

**Authors:** Chao Nie, Chao Xu, Zhengping Li, Lingling Chu, Yunxue Hu

**Affiliations:** 1 School of Integrated Circuits, Anhui University, Hefei 230601, China; 2 Anhui Engineering Laboratory of Agro-Ecological Big Data, Hefei 230601, China

**Keywords:** specular reflection, highlight detection, image inpainting, image enhancement, endoscopic image

## Abstract

Specular Reflections often exist in the endoscopic image, which not only hurts many computer vision algorithms but also seriously interferes with the observation and judgment of the surgeon. The information behind the recovery specular reflection areas is a necessary pre-processing step in medical image analysis and application. The existing highlight detection method is usually only suitable for medium-brightness images. The existing highlight removal method is only applicable to images without large specular regions, when dealing with high-resolution medical images with complex texture information, not only does it have a poor recovery effect, but the algorithm operation efficiency is also low. To overcome these limitations, this paper proposes a specular reflection detection and removal method for endoscopic images based on brightness classification. It can effectively detect the specular regions in endoscopic images of different brightness and can improve the operating efficiency of the algorithm while restoring the texture structure information of the high-resolution image. In addition to achieving image brightness classification and enhancing the brightness component of low-brightness images, this method also includes two new steps: In the highlight detection phase, the adaptive threshold function that changes with the brightness of the image is used to detect absolute highlights. During the highlight recovery phase, the priority function of the exemplar-based image inpainting algorithm was modified to ensure reasonable and correct repairs. At the same time, local priority computing and adaptive local search strategies were used to improve algorithm efficiency and reduce error matching. The experimental results show that compared with the other state-of-the-art, our method shows better performance in terms of qualitative and quantitative evaluations, and the algorithm efficiency is greatly improved when processing high-resolution endoscopy images.

## 1. Introduction

**Figure 1:**
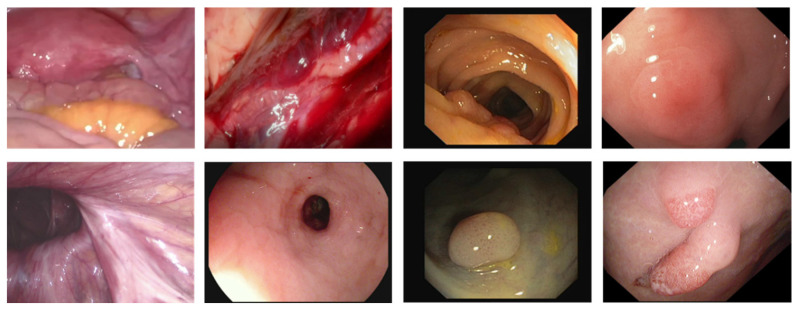
Endoscopy images with specular highlights.

With the rapid development of digital diagnosis and treatment technology, minimally invasive surgery (MIS) has been widely used in medical treatment [1], it has the advantages of small trauma areas, small physical damage to patients, and a low probability of complications. Many minimally invasive surgery is guided by the optical imaging system. An endoscope is the most commonly used method for image-guided minimally invasive surgery. However, the quality of images and/or videos provided by endoscopes often deteriorates due to the existence of specular reflection [2,3]. In the endoscopic image, specular reflection phenomena generally occur. This situation is shown in Figure 1. This is because the distance between the camera light and the surface of the organs is relatively close, the surface of the organs is smooth and humid, and the texture is sparse. The mucosa surface in the organs often produces strong specular reflections. It may block important information related to organs and appear as additional features. This not only has a negative impact on visual quality but also significantly affects the results of the follow-up computer vision/image processing algorithm. Specifically, it may potentially affect the doctor’s visual experience, deviate the attention of doctors from actual clinical goals, and hinder the tracking of surgical equipment [4]. In addition, through vaginal mirror screening for cervical cancer, specular reflection may change the appearance of the tissue and interfere with the performance of the segmentation of the cervical lesions area algorithm [5,6,7]. In colonoscopy imaging research, the detection of polyps and colorectal cancer may be affected by specular reflection [8,9]. Laryngeal cancer is detected by the laryngeal mirror, and the interference caused by specular reflection may cause the correct classification of laryngeal cancer tissue to fail [10]. Therefore, in medical imaging applications, while retaining the original color and texture details of the organ, removing specular reflection has great research significance and medical value.

Many researchers have tried to solve the problem of specular reflection in endoscopic images from two perspectives of highlight detection and highlight removal. Color space-based methods [11,12,13,14,15,16,17,18,19,20] mainly detect highlights by thresholding different color channels, which usually require empirical threshold setting, and endoscopic images in different scenes may require a large number of parameter searches. Most of these methods focus on processing mid-brightness endoscopic images. On the one hand, they are less robust to some faint highlights in dark areas and complex textures. On the other hand, for high-brightness images, they may entire high-brightness area in the image is regarded as a highlight, and the false detection rate is high. Filter-based methods [21,22] complete the center point reconstruction by designing filters to extract neighborhood information. It needs to set reasonable empirical parameters, which may be effective for small areas of highlights, but often only half of the restoration can be completed for larger areas of highlights. Two-color reflection model-based methods [23,24] utilize intensity ratios to separate the specular component and diffuse reflection in an image. It has a certain effect on the highlight removal of natural images, but the restoration performance of endoscopic images is relatively poor, and the restored images will produce problems such as discordant color distortion. The RPCA method [20,25,26] obtains the low-rank matrix and sparse matrix by PCA iterative decomposition of the image, measures the similarity between the sparse matrix and the detected highlight matrix to end the iteration, and uses the low-rank matrix to repair the specular region. the highlights can no longer be considered sparse parts, and the method does not work well. Image inpainting-based methods [6,7,8,19,27,28,29,30,31] first detect the highlighted regions in the image and then inpaint with the most similar parts in the image. It can restore the specular regions under certain conditions, but it may be unreasonable for the reconstruction of human tissue structure, or the recovery effect for large-area specular regions is not good, or the algorithm operation efficiency is low. Deep learning-based methods [4,32,33,34] usually achieve high highlight detection accuracy and reasonable restoration effects, but they suffer from high computational complexity. In addition, most of these methods require a large amount of training data, and it is difficult to find enough samples to meet the demand for actual training.

Existing technologies mainly face several challenges. First, they have high computational complexity and are not suitable for real-time processing and applications. Second, they have a poor ability to detect highlights of high-definition endoscopic images with different brightness. Third, none of these studies attempted to enhance low-light images while removing highlights. Fourth, the existing highlight removal methods cannot effectively balance color, texture, structural information preservation capabilities, and operating efficiency, and cannot better meet the follow-up computer vision algorithms and clinical diagnosis.

In this study, we propose a detection and inpainting algorithm for specular reflection in endoscopic images based on brightness classification, which can alleviate the above challenges. First of all, in terms of highlight detection, we choose the method based on color space instead of the deep learning method, which is more advantageous in practice, and solve the problem that this method cannot adaptively adjust parameters and image brightness in different scenarios. Secondly, in terms of highlight restoration, we have improved the exemplar-based algorithm with a better restoration effect, which can not only better preserve the integrity of image texture and continuity of structure, but also greatly shorten the image restoration time. The technical contributions of this paper are as follows:

- We design an image brightness classification and low brightness image brightness enhancement algorithm. This helps the follow-up highlight detection algorithm to achieve better results, and improves the image quality of low-light images;
- We develop an adaptive thresholding algorithm based on image brightness. It removes the barriers of color space-based methods;
- We propose an exemplar-based inpainting algorithm using an adaptive search range. Its advantage is that it not only reduces the error matching, but also solves the problem of extremely low algorithm efficiency as the image resolution increases, and is suitable for different high-definition endoscopic MIS procedures.

## 2. Related Works

### 2.1. SR Detection

Oh et al. [11] divided the specular reflection area into two areas: absolute highlights and relative highlights, and used region segmentation and outlier detection to complete the positioning of relative highlights. However, for images with complex textures, region segmentation algorithms often It is difficult to achieve the desired effect. The specular region segmentation method proposed by Arnold et al. [12] includes two independent modules. The first module uses a color balance adaptive threshold to determine the high-intensity highlights, and the second module converts each given pixel Compared to the smoothed non-specular color after linear filtering to detect parts of the image with weak specular highlights. Meslouhi et al. [13] proposed a method to automatically detect specular reflections in colposcopy images. They first used nonlinear filters for reflection enhancement and then calculated the brightness difference between CIE-XYZ and RGB color spaces to detect the specular reflection. In the context of surface reconstruction, Al-Surmi et al. [14] used a single grayscale threshold to detect specular reflections on the surface of the heart. Marcinczak et al. [15] proved that the accuracy of mirror detection using a single threshold technique is limited, and they proposed a more reliable hybrid detection method based on closed contours and thresholding. Alsaleh et al. [16] combined color intensity attributes based with wavelet transform-based edge projection for cardiac image highlight detection. Kudva et al. [5] combined the saturation (S) component of the HSV color space, the green component (G) component of the RGB color space, and the brightness (L) component of the CIE-Lab color space to obtain the feature image, and then uses a fixed threshold value on the output image of the filter to detect the specular reflection area of the cervical image. Gao et al. [32] considered the segmentation of specular reflection areas as a binary classification problem and used the SVM machine learning algorithm to distinguish highlighted and non-highlighted areas. Sánchez et al. [33] proposed an SVM-based colonoscopy video highlight detection method. The algorithm first obtained connected regions through a region-growing algorithm and then used an SVM classifier to filter out non-highlight regions. Recently, Alsaleh et al. [18] proposed an automatic specular suppression framework ReTouchImg, which designed an adaptive threshold function with fixed parameters based on the RGB color change of the image to detect global highlights, and combined with gradient information to detect local highlights. This method is suitable for medium-brightness images, and the detection performance is poor when the overall brightness of the image is higher/lower overall brightness. Shen et al. [19] first converted the endoscopic image into a grayscale image, then detected the highlighted region based on the empirical grayscale threshold, and then expanded the mask region using a morphological algorithm. However, this method is only suitable for some endoscopic images with uniform brightness. Li et al. [20] first eliminated the inconsistency of light and shade in the image based on the Sobel operator and then designed two adaptive threshold functions tv and ts based on the mean and standard deviation for the S value and V value of the HSV color space to extract the endoscope Image highlight pixels. Although this method has a low time complexity and is suitable for real-time processing and applications, it will ignore the detection of relatively complex highlight pixels, and the result of highlight detection is incomplete. Asif et al. [35] et al. used the Intrinsic Image Layer Separation (IILS) technique to locate the specular reflection area; however, for highly saturated, high-resolution images, the edges and highly saturated areas of the image are also misdetected as highlights.

### 2.2. SR Removal

Oh et al. [11] replaces the detected pixel value of each specular reflection area with the average intensity outside the specular reflection area to achieve the purpose of removing specular reflection. Arnold et al. [12] replaced the mirror pixels with “smooth non-reflective area color pixels”, and then used Gaussian blur to restore the image. Meslouhi et al. [13] used a multi-resolution image inpainting technique to process the specular reflection area of the colposcope. Shen et al. [23] proposed a single-image highlight removal scheme based on a two-color reflectance model, using intensity ratios to separate diffuse and specular components. Gao et al. [32] propose a method based on multi-scale dynamic image expansion and fusion to recover highlight regions and propagate regions with similar structural features to highlight regions. Funke et al. [4] utilized CNN and GAN to remove highlights in endoscopic images. Ali et al. [34] developed a deep-learning framework for detecting and suppressing highlights in endoscopic images. Their framework is built on top of Faster R-CNN, RetinaNet, and other models. Although the framework outperforms existing methods, its inference time is relatively high. Alsaleh et al. [18] adopted a graph complement method based on graph data structure to restore specular region information. Li et al. [20] propose a method based on adaptive robust principal component analysis (RPCA) to fill the specular area. Asif et al. [35] used an image fusion algorithm for endoscopic specular reflection suppression. These methods are only effective for endoscopic images with very small, highlighted regions, and texture details in inpainted regions tend to be lost. Recently, Wang et al. [6] used an exemplar-based image inpainting algorithm to restore the highlight regions of cervical images. Shen et al. [19] first classify the reflective area, and then adaptively adjusts the parameters in the exemplar-based inpainting algorithm by analyzing the image content information. The exemplar-based image inpainting algorithm can preserve the details of organ surface texture, and the repair effect is better, but the algorithm operation efficiency is too low, especially for high-resolution endoscopic images.

### 2.3. Image Inpainting

Criminisi et al. [27] proposed a classic exemplar-based inpainting method, which uses the boundary information of the damaged area to determine the priority of the sample block to be repaired, and finds the sample block most similar to the area to be repaired in the image source area to fill the target area. Based on this, Yin et al. [28] proposed a more reasonable priority function by combining the curvature and color information of pixels. In order to suppress the rapid decline of the confidence term, Jing et al. [29] introduced a regularization factor in the confidence term to adjust the priority function and combined the modified color sum of squared difference (SSD) and normalized cross-correlation (NCC) Search for the best matching block to improve matching accuracy. In order to improve the efficiency of algorithm restoration, Hui-Qin et al. [31] classify the sample blocks in the known area of the image to increase the speed of searching for matching blocks. These sample block-based methods all search for the best matching block globally, which is not only prone to mismatch, but also causes the algorithm running time to be proportional to the image resolution, and the algorithm is inefficient.

## 3. Proposed Method

Since the proposed adaptive threshold function assigns different weights to images of different brightness, it can effectively avoid the failure of the adaptive threshold function with fixed parameters: the entire area with high brightness is regarded as a highlight, and the detection of areas with low brightness is invalid, And the Sobel filter image extracted by gradient operation can also detect relative highlights. Since endoscopic images are characterized by large global variations. Our proposed exemplar-based inpainting algorithm recovers the missing information in the highlight region by utilizing the local conditions of the image: the proposed adaptive search range strategy limits the search range of the matching block to the vicinity of the highlight region and utilizes the information around the highlight region instead of using the entire image as usable information. Not only does this reduce false matches, but the running time of the algorithm is also greatly reduced and does not increase with image resolution. The proposed sequential repair strategy can limit the priority calculation range to a single highlight boundary instead of the global highlight boundary, which reduces the number of priority calculations of highlight boundaries and improves the algorithm’s efficiency.

**Figure 2:**
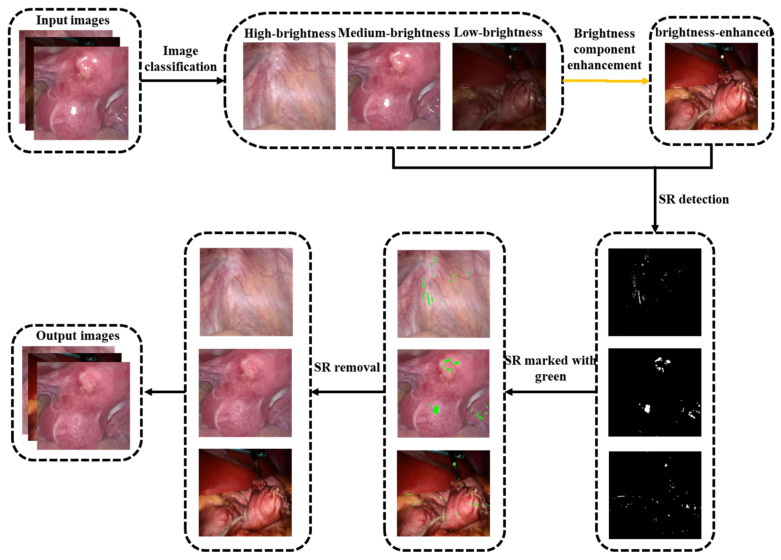
Schematic of the proposed method.

Our proposed method consists of four modules. The first part is image brightness classification. The original endoscopic image is classified according to the average brightness, and three types of images are obtained: high-brightness, medium-brightness, and low-brightness (see Section 3.1 for details). The second part is the brightness enhancement of low-brightness images. Discrete wavelet decomposition is performed on the original and adaptive gamma-corrected V channels, and singular value equalization is performed on low-frequency components to obtain brightness-enhanced images (see Section 3.2 for details). The third part is the detection of specular reflection areas. First, the image contrast is enhanced by high-hat filtering and low-hat filtering, and then the adaptive threshold function is set by using the g and b channel color changes of the enhanced image and then combined with the gradient information obtained by Sobel filtering. The highlights are jointly detected, and finally, the binary image is enlarged using the morphological dilation operation. (see Section 3.3 for details). The fourth part is the restoration of the specular reflection area. First, calculate the priority of the read single highlight area boundary, determine a 9 × 9 target block to be repaired at the pixel with the highest priority, calculate the search range X of the specular area, search for the best matching block in X and fill it in, and repair it in a loop until the repair is completed (see Section 3.4 for details). The flowchart of the proposed method is shown in Figure 2.

### 3.1. Image Classification

Due to the large brightness difference in the endoscopic image data obtained from different types of medical scanners, for example, the overall brightness of the image is relatively dark due to the complex organ structure of the stomach; some organs and lesions with relatively smooth structures, the overall image is relatively bright. This will have a great impact on the subsequent specular reflection detection algorithm. Therefore, we propose to divide the image into three categories: high-brightness, medium-brightness, and low-brightness. We set the threshold based on the average brightness of the image for image classification, and the classification rules are as follows:

(1) $$I=\left\{\begin{array}{c} I_{h}\;T_{1}>t_{1}\;\\ I_{m}-t_{1}<T_{1}<t_{1}\\ I_{l}\;T_{1}<t_{1} \end{array}\right.$$

(2) $$T_{1}=\frac{l_{a}-L_{a}}{l_{a}}$$

**Figure 3:**
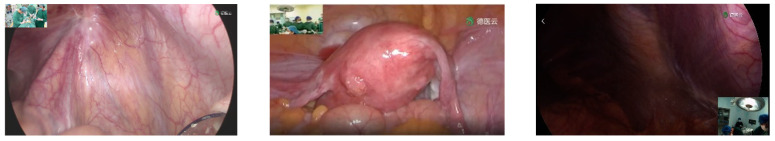

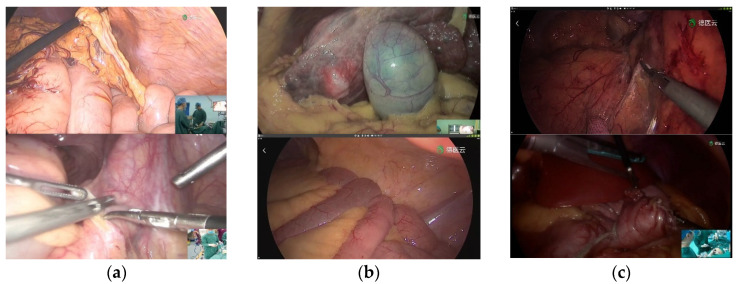
Image Brightness Classification Results. (**a**) high-brightness images; (**b**) medium-brightness images; (**c**) low-brightness images.

Among them, I is the input original endoscopic image, $l_{a}$ is the average brightness of the image, $L_{a}$ is the global average brightness of the expected normal image, $I_{h},I_{m},I_{l}$ represent high-brightness, medium-brightness, and low-brightness respectively. After a lot of experiments, it is found that when the $L_{a}$ value is 112 and the $t_{1}$ value is 0.3, the classification effect is the best. The image brightness classification results are shown in Figure 3.

### 3.2. Brightness Component Enhancement

**Figure 4:**
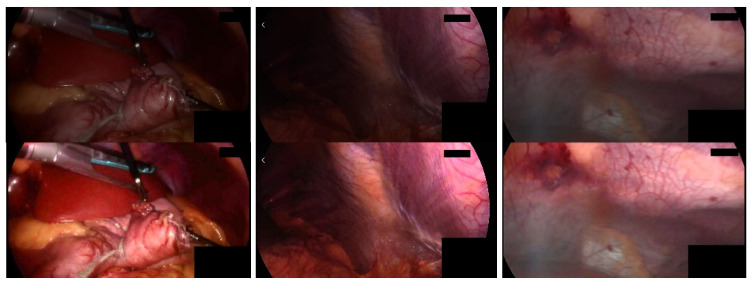
Low-brightness image brightness enhancement results. The first row is the original low-brightness image, and the second row is the brightness-enhanced image.

For low-brightness images, the brightness needs to be adjusted to enhance the visualization of its important features. To prevent over-enhancement, we perform brightness correction by performing adaptive gamma correction [36] on the V channel in HSV color space, avoiding image color distortion by preserving the hue (H) and saturation (S) channels. Singular value decomposition (SVD) [37] is considered to solve the lighting problem, the singular value matrix represents the intensity information of the input image, and any modification to the singular value matrix will affect the intensity of the image. Then we perform brightness enhancement based on the method proposed by Palanisamy et al. [38]. First, extract the V channel component, perform adaptive gamma correction on it, and obtain the low-frequency component LF_γ; use singular value decomposition to extract the low-frequency component LF of the original V channel and the singular value matrix ∆ and ∆_γ corresponding to the low-frequency component LF_γ of the gamma correction channel V_γ; calculate the equalized singular value matrix; perform inverse singular value decomposition on the equalized singular value matrix to generate a balanced low-frequency component LF_eq; use a soft threshold to denoise the high-frequency component of the original V channel, and use the low frequency equalized by the singular value The component LF_eq and the denoised high-frequency component HF perform inverse wavelet transform to obtain the enhanced V channel component of the original endoscopic image. The details of this method are discussed in the literature [38]. Figure 4 shows the brightness enhancement results. Compared with the original image in the first row, the overall brightness of the second row of images has been enhanced, and the image quality has been improved.

### 3.3. SR Detection

**Figure 5:**
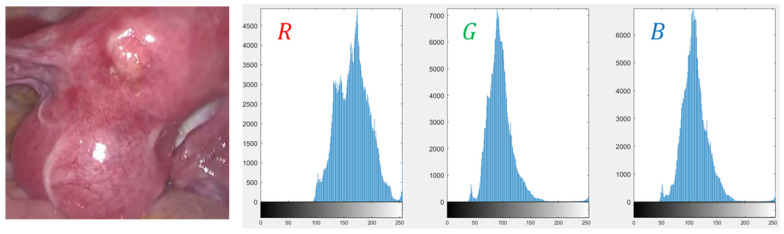
Histogram results. From left to right are the original image; red component histogram; green component histogram; blue component histogram.

For specular detection, technically speaking, highlights can be divided into absolute highlights and relative highlights [11]. Absolute highlights are generally characterized by very high-intensity values, while relative highlights are more complex, and the gradient values of their boundaries are often high. Typically, most endoscopic images are red due to the presence of hemoglobin, and the R channel has higher values than the G and B channels. However, under the specular area, the values of the three channels are almost the same and very high. Figure 5 is the histogram of the R, G, and B channels of the endoscopic highlight image. The diffuse reflection component is often red in the endoscopic image [33], and the color of the G and B channels have a large degree of distinction between diffuse reflection and specular reflection. Considering the above information, for absolute highlight detection, the adaptive threshold function proposed in this scheme only considers the color changes of the G and B channels. First, the high-hat filter and the low-hat filter are used to enhance the contrast of the highlight image to be detected, and then Extract the G and B channel components of the contrast-enhanced image, and then calculate the standard deviation $\sigma_{g,}\sigma_{b}$ of them, respectively:

(3) $$\sigma_{g}=\sqrt{\frac{1}{n-1}\sum_{i=1}^{n}{\left(x_{i}-\mu_{g}\right)}^{2}},\;\mu_{g}=\frac{1}{n}\sum_{i=1}^{n}x_{i}$$

(4) $$\sigma_{b}=\sqrt{\frac{1}{n-1}\sum_{i=1}^{n}{\left(x_{i}-\mu_{b}\right)}^{2}},\;\mu_{b}=\frac{1}{n}\sum_{i=1}^{n}x_{i}$$

Among them, n is the number of image pixels, $x_{i}$ is the current pixel value, $\mu_{g},\mu_{b}$ are the mean values of G and B channel components respectively. The proposed adaptive threshold function Th is defined as the following:

(5) $$Th=\max\left(g,b\right)-\tau \times \frac{\sigma_{g}+\sigma_{b}}{2}$$

(6) $$\tau =\left\{\begin{array}{c} 0.3\;I=h_{d}\\ \;0.8\;I=m_{d}\\ \;1.1\;I=lv_{d} \end{array}\right.$$

In Equation (5), $\max\left(g,b\right)$ is the maximum value of the intensity of the *g* and *b* channels, and *τ* is a weight parameter adaptively adjusted according to the image brightness. When the overall brightness of the image is high, the adaptive threshold function with fixed weight will cause the threshold to be too small, and more non-specular areas with high brightness will be mistakenly detected as highlights; when the overall brightness of the image is low, the adaptive threshold function with fixed weight The threshold function will cause the threshold to be too large, and more correct highlighted pixels will be missed. Therefore, for high-brightness images, the adaptive weight should be set smaller to ensure a larger threshold, and for low-brightness images, the adaptive weight should be set larger to ensure a smaller threshold. After a large number of statistical experiments, it is determined that the τ of high brightness, medium brightness, and brightness enhanced low brightness images are 0.3, 0.8, and 1.1, respectively, which are the most suitable. Equation (5) can adapt to any change in brightness of the endoscopic image, which adaptively generates a threshold in the image.

Although relative highlights have unstable saturation, chroma, and intensity values, they have drastic changes in brightness relative to surrounding pixels, so they are suitable for extraction by gradient operations. The Sobel filter map is calculated by the gradient, and under the condition of a custom threshold, the relative highlight is further refined and detected. Due to the halo effect, there may be black circles or colored circles around the bright spots. We use the morphological dilation operation to expand the detected highlight area, and the structural element used is a disc shape.

**Figure 6:**
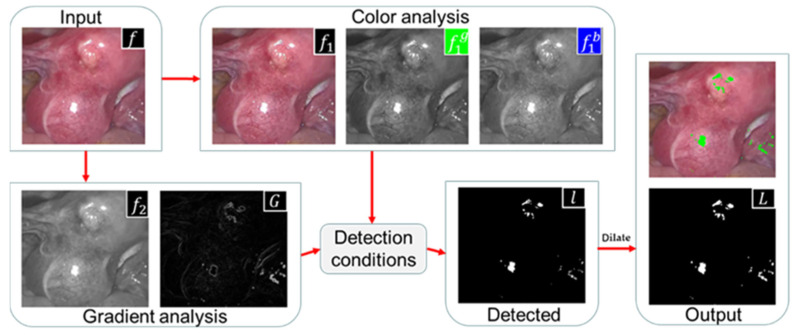
Scheme of our detection approach.

Figure 6 illustrates the flow of our detection method. In terms of color information analysis, we enhanced the contrast of the image, and extracted absolute highlight pixels by using the color change of the G and B channels; in terms of gradient information analysis, we converted the original image into gray space and used the Sobel filter to extract its gradient image. Relatively bright pixels are extracted from the gradient map. Combine the absolute highlight and relative highlight to obtain the highlight binary mask l. Finally, use the morphological expansion algorithm to expand the specular area, output the highlight detection result L, and mark the specular region in green in the original image.

### 3.4. SR Removal

Since the highlights in endoscopic images are pure specular reflections, the shapes and sizes of highlights are irregular, and the recovery performance of existing algorithms is not good. The exemplar-based algorithm [27] has good reconstruction ability for large damaged areas, but it still has the following defects in solving the problem of endoscopic highlight area repair: it may generate wrong filling and the re-pair efficiency is low. Our goal is to speed up the processing speed of the algorithm while preserving important information such as the texture and structure of the specular area.

#### 3.4.1. Exemplar-Based Algorithm

**Figure 7:**
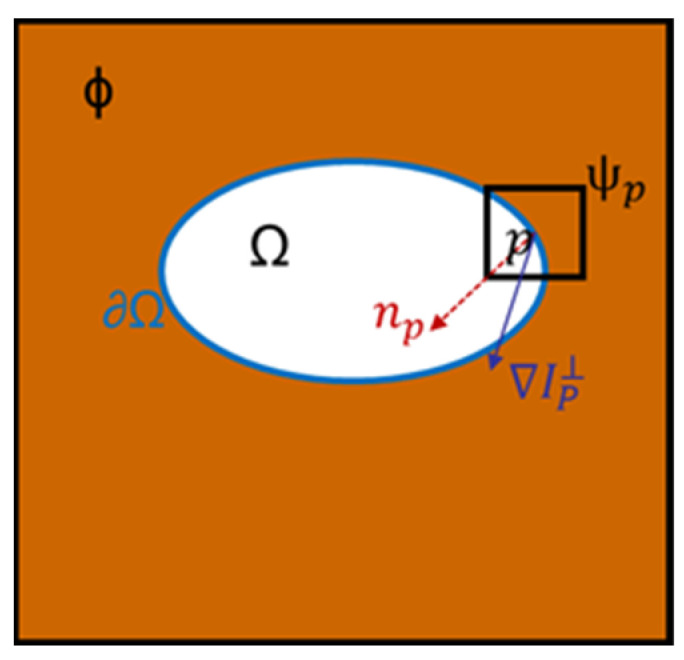
Schematic of the exemplar-based algorithm.

The schematic diagram of the exemplar-based algorithm [27] is shown in Figure 7. ϕ indicates the non-highlight area, Ω indicates the specular region to be repaired, ∂Ω indicates the boundary of the specular region, point p is the pixel with the highest priority on ∂Ω, and $\psi_{p}$ is point *p* is the centered 9 × 9 target block, $n_{p}$ and $\nabla I_{P}^{⊥}$ are the unit normal vector and isotonic line vector at point p, respectively. The Criminisi algorithm is mainly divided into the following three steps:

- Calculate boundary priority: The priority of the highlight boundary pixels determines the repair order of the sample blocks. The priority function $P\left(p\right)$ is defined as the following:

(7) $$P\left(p\right)=C\left(p\right)D\left(p\right)$$

(8) $$C\left(p\right)=\frac{\sum_{q\epsilon \psi_{p}\cap \phi }C\left(q\right)}{\left|\psi_{p}\right|}\;,0\leq C\left(p\right)\leq 1$$

(9) $$D\left(p\right)=\frac{\left|\nabla I_{P\cdot n_{p}}^{⊥}\right|}{\alpha },0\leq D\left(p\right)\leq 1$$

- In Equation (7), $C\left(p\right)$ is the confidence item, and $D\left(p\right)$ is the data item. $C\left(p\right)$ reflects the ratio of known information to unknown information in the sample block, the larger the value of $C\left(p\right)$, the more known information, $D\left(p\right)$ is used to measure the structural information at pixel point p, The larger the value of $D\left(p\right)$, the clearer the structure of the sample block.
- global search: After finding the pixel point p with the highest priority on the highlight boundary, determine the sample block $\psi_{p}$ to be repaired with point p as the center. Search the sample block globally in the image to find the matching block $\psi_{Q}$ most similar to the target block $\psi_{p}$ to be repaired. The similarity matching criterion is the SSD distance $d\left(\psi_{p},\psi_{q}\right)$:
  (10) ΨQ=argdΨq∈ϕmin(ψp,ψq) 
  (11) $$d\left(\psi_{p},\psi_{q}\right)=\Sigma \left[{\left(R_{\psi_{p}}-R_{\psi_{q}}\right)}^{2}+{\left(G_{\psi_{p}}-G_{\psi_{q}}\right)}^{2}+{\left(B_{\psi_{p}}-B_{\psi_{q}}\right)}^{2}\right]$$
- In Equation (11), *R*, *G*, and *B* represent the intensity values of each color channel. The smaller the SSD value, the higher the similarity between the target and matching blocks.
- Update confidence: After copying the corresponding area in $\psi_{Q}$ to $\psi_{p}$ to fill the area corresponding to $\psi_{p}\cap \phi$, ∂Ω must be updated, and the confidence item of the corresponding pixel must also be updated. Repeat the above steps in a loop until all the specular areas in the image are restored.

#### 3.4.2. Improved Exemplar-Based Algorithm

**Figure 8:**
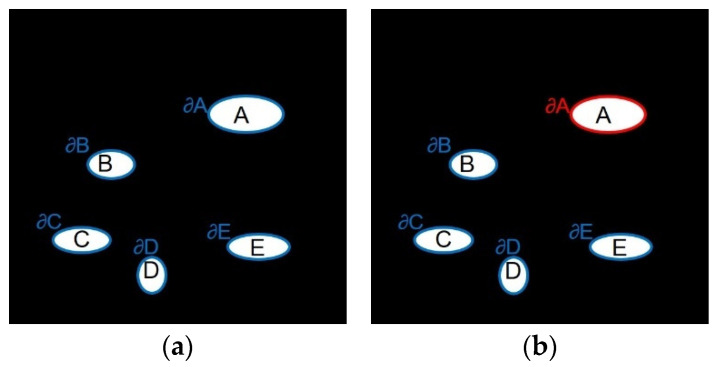
Priority calculation range. (**a**) Before improvement (blue boundary); (**b**) After improvement (red boundary).


- Local priority calculation: Figure 8 shows the calculation range of highlight boundary priority, {A, B, C, D, E} is the specular region, {∂A,∂B,∂C,∂D,∂E} is the corresponding highlight boundary, The original exemplar-based algorithm will find the boundary of the global highlight area (blue boundary in Figure 8a), by calculating the priority value of the pixel point on each highlight boundary to find For the sample block with the highest priority, the image repair sequence may jump from highlight A to another highlight B, and each time it is necessary to compare the priority of the global boundary pixels. This global calculation method may be effective for a single damaged area, but in the endoscopic image, the damaged area is generally multiple freely distributed highlights, and there is no correlation between different highlight areas. It is completely unnecessary when repairing a highlight Consider another issue of prioritization of highlights. Therefore, in the repair process, this paper only repairs one highlight at a time, and then repairs the rest of the highlights in the image based on the highlight boundary set {∂A,∂B,∂C,∂D,∂E} until the repair is completed. For example, when inpainting highlight A, only the priority of pixels on the current highlight A boundary ∂A (red boundary in Figure 8b) needs to be calculated for determining a target block, rather than the global highlight boundary. Only when the A highlight repair is completed, can the other highlight be repaired, and so on. This can reduce the number of priority calculations and shorten the running time of the algorithm without reducing the repair effect.
- Improved priority function: The original exemplar-based algorithm determines the repair order of the image according to the priority function. This function will cause two problems in the actual repair: (1) The confidence item drops sharply and tends to 0 quickly, resulting in the random selection of repair sample blocks in the repair process. To avoid this phenomenon, Jing [29] introduced a regularization factor to control the smoothness of the confidence curve in the confidence item, and Ouattara [30] changed the multiplicative definition of the priority function to a weighted summation. (2) When the confidence term is large, the data term may be zero. To solve this problem, Yin [28] added a curvature term to the data term. To sum up, this article defines the new priority function calculation formula as follows:

(12) $$P\left(p\right)=\left\{\begin{array}{c} \partial R_{C}\left(p\right)+\beta D\left(p\right)\;C\left(p\right)<0.5\\ C\left(p\right)\left[D\left(p\right)+\frac{1}{\left|K\left(p\right)\right|}\right]\;C\left(p\right)\geq 0.5 \end{array}\right.$$

(13) $$R_{C}\left(p\right)=\left(1-w\right)C\left(p\right)+w\;(0<w<1)$$

(14) $$K\left(p\right)=\nabla .\left[\frac{\nabla I_{p}}{\left|\nabla I_{p}\right|}\right]$$

In Equation (13), w represents the regularization factor that controls the smoothness of the confidence curve, and $K\left(p\right)$ is the curvature term. When $C\left(p\right)<0.5$, the new priority function can prevent the confidence term from being too low to cause the data term It is impossible to reasonably affect the priority of the specular region; when $C\left(p\right)\geq 0.5$, the confidence item has a great influence on the priority of the specular region, so continue to maintain the product form of the confidence item and the priority, and the curvature item is added to the data item, which can not only avoid the priority close to zero caused by the data item being zero but also better restore the linear structure of the image.

- Adaptive local search range: The original exemplar-based algorithm adopts a global search strategy, which means that every time a sample block is repaired, it needs to scan the whole image to find the best matching block, and the amount of calculation data is huge. The endoscopic image data is organ tissue, which is characterized by high local characteristics and significant global changes, and global information may reduce the quality of image restoration. Through a large number of statistical experiments, we found that the best matching blocks are usually distributed near the target block to be repaired. Based on the above reasons, we change the global search to an adaptive local search and adopt different expansion coefficients n to determine the matching block search area according to the contour length $l_{c}$. Expansion factor n:

(15) $$n=\left\{\begin{array}{c} n_{1},\;l_{c}<a\\ n_{2},\;l_{c}\geq a \end{array}\right.$$

Among them, a is determined by the image resolution, $n_{1}$ and $n_{2}$ are adjusted differently according to the complexity of the image texture structure, and $l_{c}$ is the length of the highlight contour. The adaptive search areas:

(16) $$u_{s}=y_{up}-nh,{\;d}_{s}=y_{down}+nh$$

(17) $$l_{s}=x_{left}-nw,{\;r}_{s}=x_{right}+nw$$

Among them, $y_{up}$ is the ordinate of the upper pole of the highlight, $y_{down}$ is the ordinate of the lower pole of the highlight, $x_{left}$ is the abscissa of the left pole of the highlight, and $x_{right}$ is the abscissa of the right pole of the highlight. Notably, w and h are the width and height of the highlight, respectively. $u_{s},d_{s},l_{s}$ and $r_{s}$ are the boundaries of the adaptive search area.

**Figure 9:**
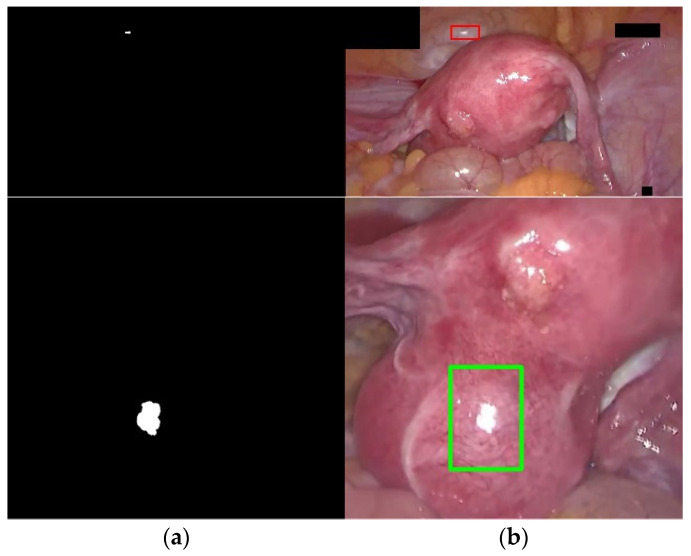
Adaptive local search. (**a**) single highlight to be repaired (**b**) search area.

Adaptive local search expands to different degrees around the highlight according to the outline length of the highlight, uses the expanded area as the search range, and assigns different search ranges to highlights of different sizes. It can be seen from Figure 9 that the adaptive local search reduces the search range so that the matching block search process is only carried out in the local area around the highlight, which can not only significantly improve the algorithm efficiency but also avoid errors caused by the global search, that is, transferring the texture of other tissues in the image to the tissue to be repaired.

## 4. Experimental Results and Analysis

The experiment in this paper uses the programming language C++ for testing. The experimental platform is the Ubuntu 20.1 x64 system, which depends on the image processing library OpenCV 4.0. The computer hardware configuration is Intel i7-7700K, 4.02 GHzx8, and the RAM is 16 GB.

### 4.1. Datasets and Evaluation

To verify the effectiveness of the proposed method for specular reflection detection and inpainting in endoscopic images, we use the endoscopic dataset DYY-Spec obtained from different types of MIS scenes and two widely used and publicly accessible datasets CVC-ClinicSpec [33] and Hyper-Kvasir [39] to evaluate, using gold standard metrics to evaluate specular reflection detection methods: Accuracy, Precision, Recall, F1-score, Dice, Jaccard, using NIQE [40] and COV to evaluate images recovery quality. The following is a brief introduction to the datasets and evaluation criteria used in this paper:

1. DYY-Spec: The DYY dataset was collected from the First Affiliated Hospital of Anhui Medical University, the Second Affiliated Hospital of Anhui Medical University, the First Affiliated Hospital of the University of Science and Technology of China, and Hefei Cancer Hospital of the Chinese Academy of Sciences. It is obtained from 4K video footage of endoscopic real surgery with a duration of about 180 min and consists of more than 5000 color images in 8-bit JPG format, including the endoscopic highlight image dataset DYY-Spec. DYY-Spec contains 1000 endoscopic specular images from data of organs with different reflective properties: uterine fibroids, bladder, groin, nasal cavity, abdominal stomach, prostate, and liver. In addition, the DYY-Spec dataset also includes specular ground truth (GT) labels annotated by experienced urologists.
2. CVC-ClinicSpec: The CVC-ClinicSpec dataset contains 612 colonoscopy images with specular ground truth labels annotated by experienced radiologists.
3. Hyper-Kvasir: Hyper-Kvasir is the largest publicly released gastrointestinal image dataset, the dataset contains a total of 110,079 images and 373 videos.
4. Highlight detection evaluation standard: In order to evaluate the segmentation effect of the detection algorithm on the high-light area, the six indicators of Accuracy, Precision, Recall, F1-score, Dice, and Jaccard are used for quantitative evaluation. Accuracy indicates the ratio of the correctly segmented pixels by the detection algorithm to the actual pixels, Precision indicates the ratio of the correct highlight pixels detected by the algorithm to the actual highlight pixels detected by the algorithm, and Recall indicates the difference between the correct high-light pixels detected by the algorithm and the actual GT image Ratio of highlight pixels. F1-score represents the comprehensive performance of the detection algorithm, which is a composite index of precision and recall and combines the results of the two. Dice and Jaccard indicate how similar the algorithm detection results are to the true results (GT). The value range of these indicators is [0, 1], the higher the value, the better the detection effect. Their calculation formulas are as follows:
  (18) $$\mathrm{Accuracy}=\frac{\mathrm{TP}+\mathrm{TN}}{\;\mathrm{TP}+\mathrm{TN}+\mathrm{FP}+\mathrm{FN}}$$
  (19) $$\mathrm{Precision}=\frac{\mathrm{TP}}{\mathrm{TP}+\mathrm{FP}}$$
  (20) $$\mathrm{Recall}=\frac{\mathrm{TP}}{\mathrm{TP}+\mathrm{FN}}$$
  (21) $$F1\text{-}\mathrm{score}=2\frac{\mathrm{Precision}\cdot \mathrm{Recall}}{\mathrm{Precision}+\mathrm{Recall}}$$
  (22) $$\mathrm{Dice}=\frac{2\mathrm{TP}}{2\mathrm{TP}+\mathrm{FP}+\mathrm{FN}}$$
  (23) $$\mathrm{Jaccard}=\frac{\mathrm{TP}}{\mathrm{FP}+\mathrm{FN}+\mathrm{TP}}$$
  Among them, TP (True Positive) indicates that the pixels on the GT label image are highlights, and the pixels on the detection result image are also highlights, and the segmentation is correct. TN (True Negative) indicates that the pixels on the GT label image are non-highlights, and the pixels on the detection result image are also non-highlights, and the segmentation is correct. FP (False Positive) means that the pixels on the GT label image are non-highlights, but the pixels on the detection result image are highlights, and the segmentation error belongs to over-detection. FN (False Positive) indicates that the pixels on the GT label image are highlights, but the pixels on the detection result image are non-highlights, and the segmentation error belongs to missing detection.

5. Highlight Removal Evaluation Criteria: Considering that the endoscopic highlight dataset lacks real highlight-free ground truth images, we use the No-Reference (NR) image evaluation metric NIQE [40] and Coefficient of Variation (COV) to quantitatively evaluate the proposed inpainting method. The no-reference image quality evaluation index refers to directly calculating the visual quality of the restored image in the absence of a reference image. The smaller the NIQE value, the higher the image quality. COV is also a no-reference image evaluation index, which indicates the intensity uniformity in the area. The smaller the COV value, the better the highlight recovery effect. The formula for calculating COV is as follows:

(24) cov=σ/μ×100

6. In Equation (24), σ and μ represent the standard deviation and standard value in the measurement area, respectively.

### 4.2. Specular Reflection Detection Results

To verify the effectiveness of the detection algorithm in this paper for the segmentation of endoscopic image highlights, this paper selects six commonly used medical image segmentation indicators for quantitative analysis and compares them with the detection results of Arnold et al. [12], Meslouhi et al. [13], Alsaleh et al. [18], Shen et al. [19], and Asif et al. [35].

**Figure 10:**
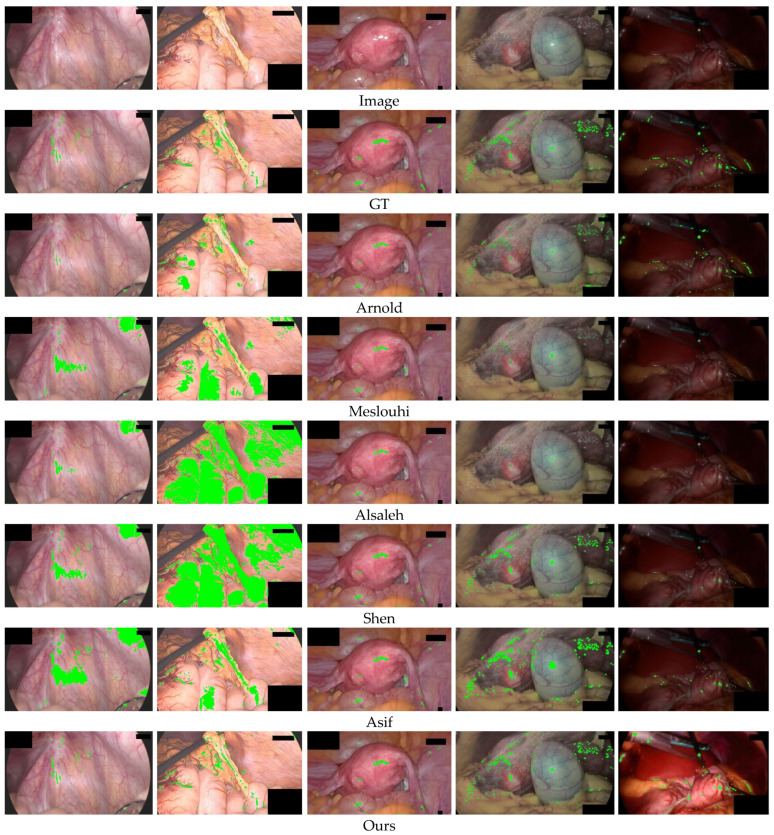
Qualitative comparison results of detection performance on the DYY-Spec dataset. From top to bottom are the original image, the specular ground truth label (GT), and the Highlight segmentation results of Arnold et al. [12], Meslouhi et al. [13], Alsaleh et al. [18], Shen et al. [19], Asif et al. [35], and Ours.

Figure 10 shows the qualitative comparison of the detection performance of different methods on the DYY-Spec dataset. The original images in the first and second columns are high-brightness images. In the example in the first column, Arnold et al. [12] cannot fully detect the highlighted area, missing several small highlights at the blood vessels, while other comparison methods will have varying degrees of over-detection, and areas with high overall brightness will be mistakenly segmented into highlights. In the second column example, the brightness difference between the highlight and the background area is small, and all comparison methods produce over-detection. Over-detection is a serious problem, which leads to the loss of original image texture details in the subsequent highlight recovery process. The original images in the third and fourth columns are medium-brightness images. In the fourth column example, Arnold et al. mis-segmented part of the yellow fat area as highlights, the results of Meslouhi et al. [13] and Alsaleh et al. [18] detected only a small fraction of specular regions, and other comparison methods only detected the absolute highlight and did not detect the relative highlight with lower brightness. In the fifth column of low-brightness images, Meslouhi et al. [13] and Alsaleh et al. [18] fail to detect, Shen et al. [19] and Asif et al. [35] can only detect part of the highlights, while our method not only accurately detected highlights but also enhanced the overall brightness component of the image, improved image quality. The visualization results show that our method outperforms the comparison methods, and the highlighted regions segmented by our method are closer to the ground truth (actual highlight regions), which leads to the highlight removal process being able to discard less ground truth information of the image.

Table 1 lists the quantitative comparison results of the detection performance of different algorithms on the DYY-Spec dataset. Among them, the values marked in red perform best in detection performance, and the values marked in green perform sub-optimally in detection performance. Compared with other methods, the values of Accuracy, Precision, Recall, F1-score, Dice, and Jaccard of our method are much better than the comparison algorithms. The Accuracy and similarity index value Jaccard of Arnold et al.’s method on the DYY-Spec dataset reached the highest among the comparison algorithms. The accuracy of the method in this paper is 1.77% higher than that of Arnold et al. [12], and the Jaccard similarity index value is 16.67% higher than that of Arnold et al. [12], which verifies the effectiveness of this method for highlight segmentation.

Table 2 lists the quantitative comparison results of the detection performance of different algorithms on the CVC-Spec dataset. Although the Recall value of our method is lower than the methods of Meslouhi et al. [13] and Alsaleh et al. [18], The proposed method has the highest F1-score value, that is, the harmonic mean of Precision and Recall is the highest, which is 3.9% higher than the second-ranked Shen et al. [19] method, and other indicators of our method higher than the comparison method. This indicates that our method has the best comprehensive performance, which further verifies the effectiveness of the highlight detection method in this paper.

**Figure 11:**
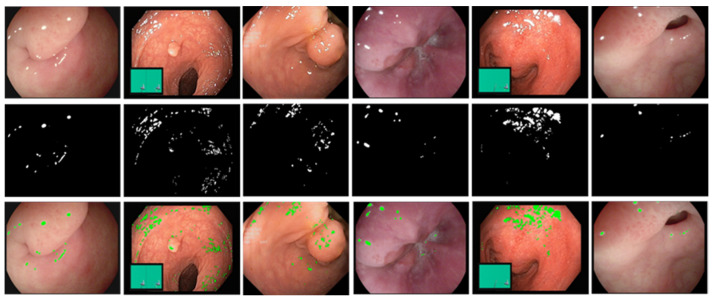
Qualitative results of highlight detection by our method on the Hyper-Kvasir dataset, first row: original highlight image, second row: highlight detection result, third row: highlight detection result label.

Furthermore, we verify the generality of our method on the Hyper-Kvasir dataset. Figure 11 shows the qualitative results of highlight detection by our method. The first row is the input frame with different reflection intensities and different percentages of highlighted pixels in the Hyper-Kvasir dataset. The second and third rows are the final result of specular reflection detection and the labeled image, respectively, and the detected highlight regions are marked in green. Observe from the third row that the green marker can successfully cover the specular region in the image. This shows that the detection method in this paper performs well on various MIS scenarios, and has a strong generalization ability.

### 4.3. Specular Reflection Suppression Results

To verify the effectiveness of the inpainting algorithm in this paper for suppressing the specular region of endoscopic images, we compared the method in this paper with the methods of Arnold et al. [12], Shen et al. [23], Li et al. [20], Asif et al. [35], Wang et al. [6], and Yin et al. [28], and selected two commonly used no-reference (NR) image evaluation indicators NIQE and coefficient of variation (COV) to quantify analysis.

**Figure 12:**
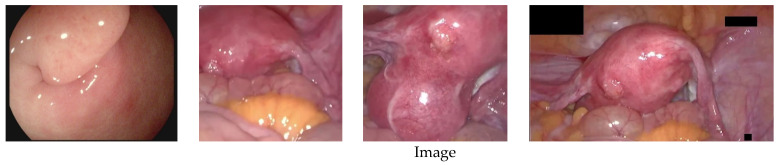

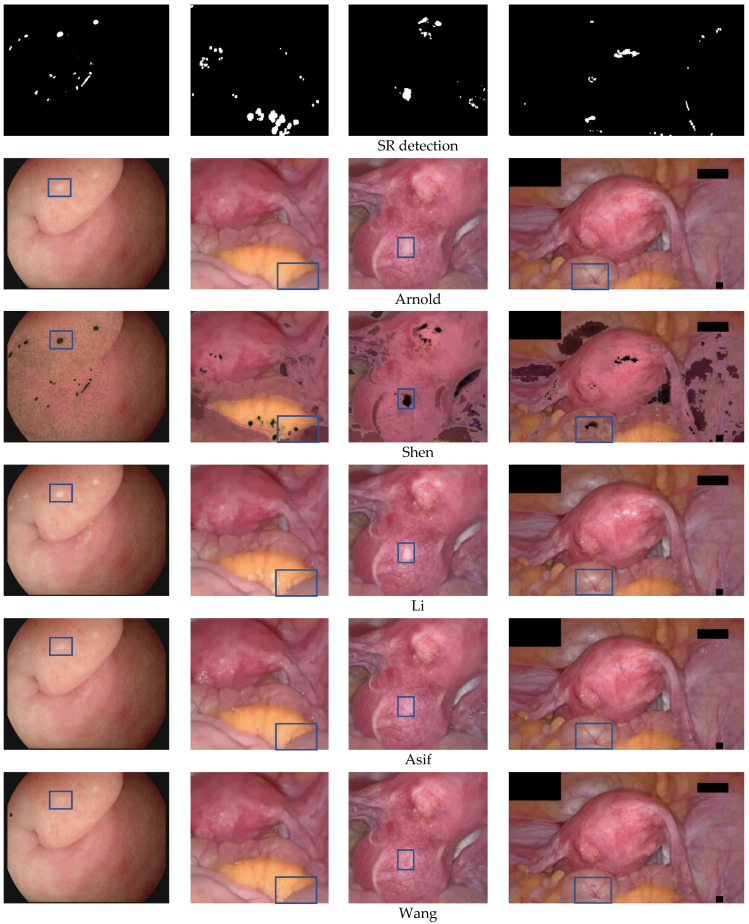

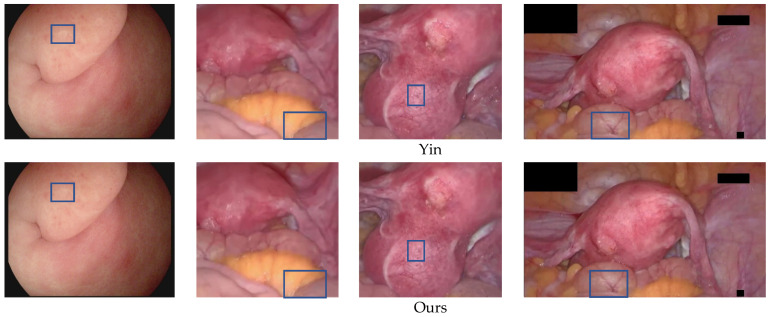
Comparison of qualitative results of highlight removal. From top to bottom are the original image, specular reflection region, and the highlight removal results of Arnold et al. [16], Shen et al. [23], Li et al. [20], Asif et al. [35], Wang et al. [6], Yin et al. [28], and Ours.

Figure 12 shows a qualitative comparison of the highlight removal performance of different methods. In the third row, the specular region recovered by Arnold et al. [12] is blurred, and the texture and detailed information behind the highlighted cannot be restored. In the fourth row, the method [23] based on the dichromatic reflection model is difficult to cleanly remove the specular reflection, and the highlight part removal results contain a large number of black spots and cause severe degradation and color distortion. In the example in the fifth row, the specular region recovered by the RPCA-based method [20] is relatively white, and cannot correctly reconstruct the texture and details of the larger highlight area. This is because the RPCA-based method is based on the image as a real low-rank case, but endoscopic images are not exactly low-rank. In the example in the sixth row, there is some texture loss in the result of the highlight region reconstructed by the state-of-the-art endoscopic image highlight removal algorithm [35]. In the first column example, there is a black spot in the inpainting result of the original exemplar-based algorithm [6], which is caused by the wrong match caused by the global search, as indicated by the blue box mark, Wang et al. [6] and Yin et al. [28] have obvious border artifacts in the restored highlight area, and both have a small highlight residue. Compared with other methods, the proposed method produces more realistic and natural inpainting results with good preservation of texture and detailed information. In the blue box marks of the examples in the second column, none of the recovery results of the comparison methods meet visual expectations, and the dividing line between yellow fat and red tissue is unreasonable. Wang et al. [6] and Yin et al. [28] can eliminate highlights, but there is a phenomenon of losing original pixel information at the boundary. However, the proposed method produces more reasonable inpainting results, which meet visual expectations. In the blue box marks of the third and fourth column examples, the results produced by Wang et al. [6] and Yin et al. [28] have content distortion, and the structural information reconstruction of blood vessels is unreasonable. Our method produces better visual results, capable of accurately reconstructing blood vessels and texture details.

To further verify the effectiveness of the highlight suppression algorithm proposed, we quantitatively compare the recovery performance of different algorithms by calculating the average of different algorithms on the average of the NIQE and COV on the 30 endoscopic images. Table 3 lists quantitative comparison results. Among them, the black thick value performed best in recovery performance. The average NIQE value of the proposed method is reduced by 0.6479, which is 0.0729 more than the original image and yin et al. [28], respectively. The average COV value of our method is reduced by 0.0285, which is 0.0102 more than the method of original images and yin et al. [28], respectively. NIQE values and COV values based on the two-color reflection model are the highest, and the highlights removal effect is very poor. Compared with other methods, the proposed method’s average NIQE and COV values have decreased to varying degrees. This means that the image quality of our method is higher, and the highlight removal effect is better.

It is worth noting that although NIQE and COV can help objectively evaluate highlight recovery performance by evaluating the quality of the image and the strength uniformity in the region, the subjective visual assessment is often more important because the ground truth of the specular regions is impossible to restore.

To verify the effectiveness of the local priority computing and adaptive local search strategy proposed in this article, in terms of the operation time of the algorithms, we compared the proposed method with the RPCA-based method [25], the exemplar-based algorithm [6], and Yin [28] et al.’s method. Table 4 shows the calculation time of different methods on the 16 endoscopic images, and our method consumes the least time. Our approach is at most 180.4 s faster than RPCA, at least 7.73 s faster. The RPCA-based method [25] requires a strange value decomposition of the image matrix. The time complexity of the odd value decomposition is O3. Calculating time is greatly affected by the image resolution. The proposed method is at most 4722.62 s faster than the original exemplar-based algorithm, at least 30.49 s faster, and the operating efficiency has increased by up to 659 times, and at least 58 times. The exemplar-based algorithm [6] must calculate the priority of the pixels of all specular regions’ boundaries every time they are inpainted, which causes the algorithm to increase many unnecessary priority calculations. More importantly, every time it repairs a sample block, it needs to scan the overall image to find the best matching block. This process consumes most of the algorithm operation time, which leads to the higher the image resolution, the larger the matching block search range, and the longer the running time. Yin [28] et al.’s method does not need to search for sample blocks that have been used once in the search process, but, the global search strategy is used, the amount of data in the search process is still very large, so it takes a long time. The method of this article only needs to calculate the border priority of the current highlight area, which can reduce the number of priority calculations and shorten the operating time of the algorithm without reducing the inpainting effect. In addition, the adaptive local search re-duces the scope of the search, the matching block search process is performed only in the local area around the highlights, which significantly improves the algorithm’s efficiency. This makes the running time only positively correlated with the number of highlight pixels and the size of the specular regions, which has nothing to do with the size of the image itself. Therefore, compared with the comparison method, this method has more advantages in terms of efficiency, especially for endoscopic images with high resolution and multiple distributed highlights.

### 4.4. Clinical Validation

In order to verify the actual significance of the repair algorithm in this article, we have performed clinical verification and compared our method with the methods of Arnold et al. [12], Shen et al. [23], Li et al. [20], Asif et al. [35], Wang et al. [6], and Yin et al. [28]. We invite 10 surgeons from Anhui Medical University to blindly evaluate the highlighted recovery results of different methods. By removing a maximum score and a minimum score, the remaining eight doctors’ average scores were calculated to attain the final score. The evaluation dataset is 30 endoscopic images from different MIS scenarios. The evaluation standards are effective, high quality, and helpful. The effect refers to the recovery effect of the endoscopic image highlight areas, it represents the ability to remove highlights. Quality refers to the image quality after highlight removal, which reflects the ability to retain the color, texture, and structural information of the image. Help refers to the ability to improve actual minimally invasive surgery. The value range of these three evaluation standards is 1–10, the higher the value, the better the clinical effect. Table 5 gives a quantitative comparison result of different methods. The scores of the three indicators in this article are the highest, and the best clinical verification results are obtained.

## 5. Conclusions

In this article, we propose a specular reflection detection and removal method for endoscopic images based on brightness classification. Based on the average brightness, the endoscope image is divided into three categories, and we use adaptive gamma correction and strange value balance to enhance the brightness component of low-brightness images. In the detection scheme, we use the G, B channel color change to set the adaptive threshold function to detect absolute highlights. This function contains adaptive weight parameters that change with the brightness of the image. Next, combined with the gradient information obtained by Sobel filtering to detect relative highlights. Finally, use the morphological expansion operation to expand the binary image. Our detection solutions overcome the inherent challenges in the existing scheme, including artificial selection parameters and changes that cannot adapt to brightness. We have also verified the advantages of absolute highlights and relative highlights. In the inpainting scheme, we use the improved Exemplar-based image inpainting algorithm to restore the texture and structure information behind the specular regions, realized the good approximation of the large specular regions, and improved the operating efficiency of the algorithm.

We conducted experimental assessments on many endoscopic datasets from different MIS scenarios. The results show that the Accuracy, Precision, F1-score, Dice, and Jaccard of our detection schemes are better than other advanced methods. Our highlight removal scheme has achieved the best visual effects, The NIQE and COV values of this method are better than the current popular highlight removal method, and the operating efficiency has been increased by dozens to hundreds of times compared to the original exemplar-based image inpainting method. It is Suitable for high-definition endoscopy images. Although the inpainting scheme in this article can be used as a pre-processing step of medical image analysis and application, it cannot be applied to real-time surgical operation scenarios. This may require hardware to accelerate, such as using Field Programmable Gate Array for parallel operations. Therefore, future work will be studied parallel operations so that they can be applied to more real-time processing occasions such as video.

## Figures and Tables

**Table 1:** Quantitative comparison results of detection performance on the DYY-Spec dataset.

Methods	Accuracy	Precision	Recall	F1-Score	Dice	Jaccard
Arnold et al. [12]	0.9672	0.4968	0.7710	0.6042	0.5135	0.6619
Meslouhi et al. [13]	0.9344	0.3725	0.7134	0.4900	0.2451	0.5394
Alsaleh et al. [18]	0.9602	0.5167	0.5780	0.5456	0.2470	0.5509
Shen et al. [19]	0.8618	0.6002	0.7143	0.6522	0.3035	0.5257
Asif et al. [35]	0.9437	0.6280	0.7079	0.6655	0.3973	0.6011
Proposed	0.9849	0.7575	0.8869	0.8171	0.7673	0.8286

**Table 2:** Quantitative comparison results of detection performance on the CVC-Spec dataset.

Methods	Accuracy	Precision	Recall	F1-Score	Dice	Jaccard
Arnold et al. [12]	0.9837	0.8938	0.6739	0.7684	0.4754	0.6589
Meslouhi et al. [13]	0.9920	0.3744	0.8961	0.5281	0.4727	0.6616
Alsaleh et al. [18]	0.9699	0.6016	0.8020	0.6875	0.4801	0.6016
Shen et al. [19]	0.9767	0.9064	0.6683	0.7694	0.4690	0.6518
Asif et al. [35]	0.9584	0.6972	0.7151	0.6489	0.4600	0.6333
Proposed	0.9932	0.9083	0.7286	0.8085	0.6084	0.7153

**Table 3:** Test results of NIQE and COV.

Methods	NIQE	COV
Original	6.4315	0.3235
Arnold et al. [12]	6.3130	0.3157
Shen et al. [23]	6.7897	0.3670
Li et al. [20]	6.2093	0.3173
Asif et al. [35]	6.1103	0.3059
Wang et al. [6]	5.9965	0.3054
Yin et al. [28]	5.8565	0.3052
Proposed	**5.7836**	**0.2950**

**Table 4:** Test results of NIQE and COV. Test results of running time(s). From left to right are the original image number, image size, the total number of pixels of the image, the highlight percentage, the number of highlight pixels, the running time results of Li et al. [20], Wang et al. [6], Yin et al. [28] and proposed.

Images	Size	Total Pix	Percentage	Highlight Pix	Li et al. [20]	Wang et al. [6]	Yin et al. [28]	Proposed
1	288 × 384	110,592	0.62	689	8.31	30.92	29.81	0.43
2	288 × 384	110,592	1.15	1276	9.97	46.01	44.34	0.68
3	288 × 384	110,592	2.00	2184	8.50	49.56	48.01	0.77
4	485 × 506	245,410	1.51	3697	17.61	164	159	1.51
5	555 × 528	293,040	1.28	3753	21.11	209	201	3.01
6	419 × 798	334,362	1.38	4630	21.49	635	613	3.75
7	602 × 753	453,306	0.72	3250	35.18	300	286	1.74
8	652 × 824	537,248	1.43	7662	37.31	1592	1570	4.86
9	950 × 930	883,500	2.61	22,940	88.46	2676	2652	45.80
10	1280 × 720	921,600	0.74	6837	64.59	1385	1349	3.17
11	1057 × 1080	1,141,560	1.01	11,505	107.31	4737	4702	14.38
12	1026 × 1155	1,185,030	0.31	3630	104.74	1146	1095	1.74
13	1011 × 1210	1,223,310	0.67	8220	128.65	2471	2455	5.49
14	1024 × 1280	1,310,720	0.55	7160	159.85	2807	2576	5.99
15	1070 × 1348	1,442,360	0.58	8340	177.94	2301	2107	10.53
16	1001 × 1516	1,517,516	1.11	16,625	191.95	4711	4673	11.55

**Table 5:** Quantitative comparison results of different methods based on the blind assessment of the surgeon.

	Arnold et al. [12]	Shen et al. [23]	Li et al. [20]	Asif et al. [35]	Wang et al. [6]	Yin et al. [28]	Ours
Effect	7.3	5.7	7.1	7.7	8.0	8.1	8.5
Quality	7.2	4.4	6.9	8.1	8.4	8.4	9.0
Help	7.2	4.1	7.3	8.0	8.1	8.2	8.6

## Data Availability

Not applicable.
